# Effects of Nitric Oxide Expression on Hearing Loss

**DOI:** 10.3390/ijms26178416

**Published:** 2025-08-29

**Authors:** Yoo Jin Cha, Joon Hyung Yeo, Sung Soo Kim, Jae Min Lee, Yeon Ju Oh, Dong Keon Yon, Seung Geun Yeo

**Affiliations:** 1 Department of Medicine, College of Medicine, Kyung Hee University Medical Center, Seoul 02447, Republic of Korea; swj09161@khu.ac.kr (Y.J.C.); 5duswn1203@khu.ac.kr (Y.J.O.); 2 Public Health Center, Danyang-gun 27010, Republic of Korea; joonhyungyeo@gmail.com; 3 Medical Research Center for Bioreaction to Reactive Oxygen Species and Biomedical Science Institute, Core Research Institute (CRI), Kyung Hee University, Seoul 02447, Republic of Korea; sgskim@khu.ac.kr; 4 Department of Otorhinolaryngology Head & Neck Surgery, Kyung Hee University School of Medicine, Kyung Hee University Medical Center, Seoul 02447, Republic of Korea; sunjaesa@hanmail.net; 5 Center for Digital Health, Medical Science Research Institute, Kyung Hee University School of Medicine, Kyung Hee University Medical Center, Seoul 02447, Republic of Korea; 6 Department of Precision Medicine, Graduate School, Kyung Hee University, Seoul 02447, Republic of Korea; 7 Department of Convergence Medicine, College of Medicine, Kyung Hee University, Seoul 02447, Republic of Korea

**Keywords:** hearing loss, nitric oxide, pathogenesis, nitric oxide synthase

## Abstract

The role of nitric oxide (NO) in the onset and pathogenesis of hearing loss remains a matter of debate. To address this, we conducted a narrative review of the literature on the subject. We performed a literature search of SCOPUS, PubMed, Cochrane Library, EMBASE, and Google Scholar databases on the production and role of NO in hearing loss using the search terms/strategy “nitric oxide” AND “hearing loss” to ensure a comprehensive review of available studies. Results: Of 186 papers initially retrieved, 166 were unrelated to hearing loss and NO and were excluded. Of the 23 papers ultimately reviewed, 58% (12 articles) reported that NO caused or worsened hearing loss, 26% (5 articles) reported a beneficial effect of NO in the treatment of and/or defense against hearing loss, and 16% (3 articles) reached no firm conclusion on whether NO played a positive or negative role. This review highlights the dual role of NO in auditory health, where it is essential for normal cochlear function through regulation of blood flow and neurotransmission. However, excessive or dysregulated NO production, particularly via inducible nitric oxide synthase (iNOS), can lead to oxidative stress and hearing loss. Conversely, NO also exhibits protective effects in certain contexts, such as reducing noise-induced hearing damage through its antioxidant properties. These findings underscore the potential of NO modulation as a therapeutic strategy for hearing loss, emphasizing the need for further research to optimize its application and understand the conditions under which it is beneficial or harmful.

## 1. Introduction

### 1.1. Nitric Oxide

**Figure 1:**
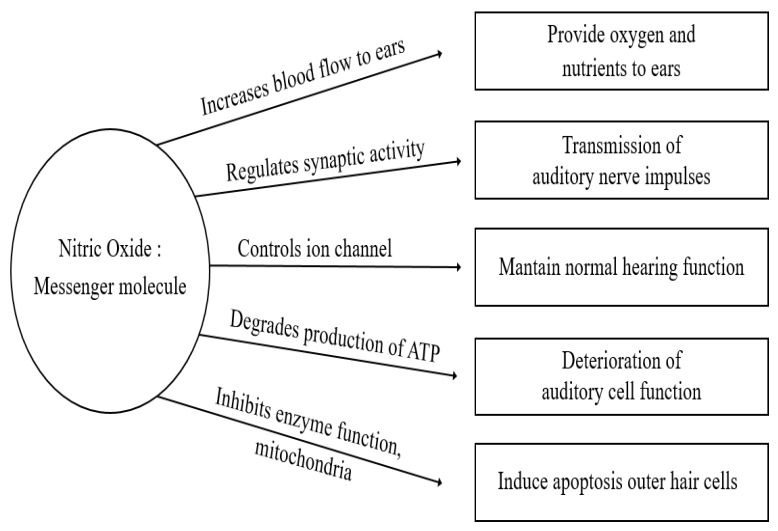
The dual roles of NO in auditory function. NO is a signaling molecule that increases blood flow to the ears to promote the supply of oxygen and nutrients, and also maintains normal auditory function through ion channel regulation. However, excessive NO inhibits enzyme function and ATP production, inducing cell apoptosis, which can degrade hearing cell function. NO protects hearing at the physiological level, but can damage hearing through oxidative stress when present in excess levels.

Nitric oxide (NO) is a free radical consisting of one oxygen and one oxidized nitrogen that is characterized by a single unpaired electron and high chemical reactivity. NO, a crucial component of signal transduction pathways involved in various physiological processes, including immune action, vasodilation, and signal transmission, is formed intracellularly from the amino acid arginine by a reaction mediated by the enzyme nitric oxide synthase (NOS) [1,2] (Figure 1). Exposure to excessive concentrations of NO is linked to various health risks, including respiratory conditions like chronic obstructive pulmonary disease and cardiovascular issues such as arteriosclerosis. It is also associated with inflammatory and autoimmune disorders, and neurological conditions like stroke and dementia due to its ability to affect blood flow and immune responses [3,4]. However, at physiological levels, NO is crucial as a signaling molecule that promotes vasodilation and supports healthy circulation, highlighting the necessity of balanced NO production for maintaining health. Disruptions in this balance, whether from overproduction or prolonged exposure, can lead to pathological conditions [5].

### 1.2. Hearing Loss

Exposure to intense noise can lead to both temporary and permanent threshold shifts in hearing, primarily due to damage to cochlear hair cells and synaptopathy. Noise-induced hearing loss (NIHL) involves the accumulation of reactive oxygen species (ROS) and reactive nitrogen species, which activate stress pathways leading to cellular apoptosis and necrosis. Key mechanisms include oxidative stress, calcium dysregulation, and inflammation, which contribute to hair cell and synaptic damage [6]. Sensory hair cells in the inner ear are continuously exposed to mechanical stress and various ototoxic factors, resulting in damage over time. This damage can manifest as dysfunction of mechanotransduction complexes, loss of ribbon synapses, and eventual hair cell death. Despite the inability of mammalian hair cells to regenerate, several mechanisms exist to repair minor damage, thereby preserving auditory function [7].

Hearing loss, as the name suggests, refers to symptoms associated with difficulty in hearing sounds. It is largely classified into three categories: conductive hearing loss, sensorineural hearing loss, and mixed hearing loss. Conductive hearing loss occurs when sound transmission is impeded along the pathway from the outer ear to the cochlea, often due to issues with sound amplification in the middle ear [8,9]. Mixed hearing loss is simply a combination of both conductive and sensorineural components. Our primary focus, however, is on sensorineural hearing loss, which is caused by damage to the cochlea’s hair cells, or abnormalities in the auditory nerve or central auditory pathways [10,11]. These issues result in impaired sound detection and transmission to the brain. Research on the relationship between NO and hearing loss has predominantly centered on sensorineural hearing loss, particularly regarding the auditory nerve or central nervous system pathways that transmit sound-induced stimuli to the brain.

NO acts as a volume transmitter in the auditory system, modulating neuronal excitability and synaptic transmission in response to auditory stimuli. While it can enhance auditory processing by affecting ion channels and synaptic activity, dysregulated NO signaling during stress or pathological conditions can contribute to hearing loss. NO’s dual effects—protective versus harmful—depend on its concentration and the affected auditory structures, such as hair cells and neurons. Understanding these mechanisms is essential for elucidating NO’s role in hearing loss pathogenesis [12]. Some studies suggest that NO has protective effects against NIHL due to its antioxidant properties, while others indicate that NO exposure can exacerbate NIHL by causing irreversible structural changes to outer hair cells or impairing their responsiveness, especially during infections. In light of these conflicting findings, we conducted a systematic literature review to examine in detail the role of NO in the onset and recovery of hearing loss.

## 2. Research Methods

**Figure 2:**
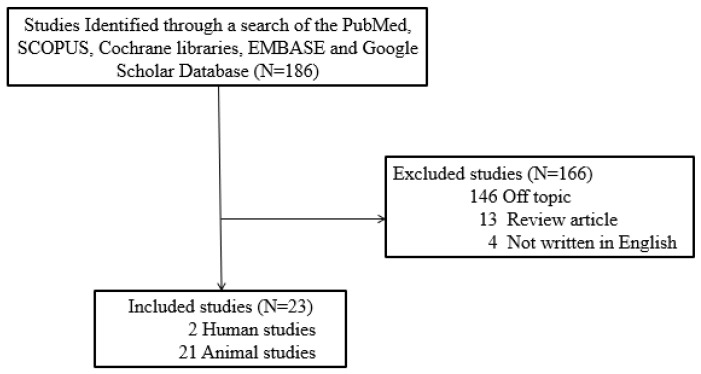
Review flow diagram. Schematic showing the systematic literature search on the subject of the relationship between NO and hearing loss.

Although there have been reviews on the role of NO in a number of diseases, there has not been a comprehensive review of the literature on the production and role of NO in the development of hearing loss. To address this gap in our knowledge, one author (Y.J.C) searched for studies published between January 1997 and December 2024 in five electronic databases—Cochrane Libraries, EMBASE, Google Scholar, PubMed, and SCOPUS—using the search terms/strategy ‘hearing loss’ AND ‘nitric oxide’. For inclusion in our review, studies needed to meet several criteria. Firstly, they had to be published in English to ensure accessibility and uniformity in language. We included both prospective and retrospective studies that specifically examined the role of NO in hearing loss, whether conducted on human or animal subjects. Regarding sample sizes, while we did not set a strict minimum threshold, we prioritized studies with larger sample sizes to ensure statistical robustness. We also required studies to use standardized criteria or measurements, such as audiometric thresholds, to define hearing loss. For studies related to noise-induced hearing loss, we included only those that provided detailed descriptions of their noise exposure protocols. This included specifying the intensity, duration, and type of noise exposure, as these details are crucial for understanding the impact of noise on hearing loss and ensuring comparability across studies. Inclusion criteria also encompassed studies that examined exposure to occupational noise alone or in combination with other factors, and those that assessed hearing loss and other health outcomes, with a statistical association between occupational noise and hearing loss/other health outcomes. Our exclusion criteria were designed to maintain the quality and relevance of our review. We excluded unpublished data, review articles, gray literature, case reports, and duplicates. Out of the 186 studies initially identified, 146 were excluded for being off-topic, 13 were review articles, and 4 were not published in English. Consequently, 23 studies met our criteria and were included in our review (Figure 2). The literature search was designed to focus on preclinical studies, primarily animal models, investigating the effects of NO on hearing loss. Cellular and human studies in this field are limited, but animal models offer valuable insights into the efficacy and mechanisms of NO.

## 3. Studies on the Role of NO in Hearing Loss

**Figure 3:**
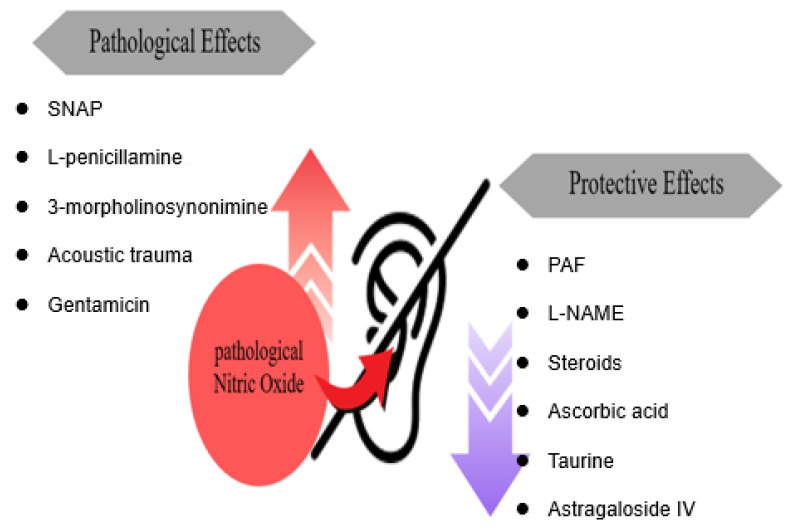
The mechanism by which increased NO contributes to the pathogenesis of hearing loss. Five substances have pathological effects that induce hearing loss by increasing NO. In contrast, six substances with protective effects safeguard the ear from hearing loss by inhibiting NO.

To resolve the role of NO in the pathogenesis of hearing loss, we analyzed 20 related research results. Twelve papers reported that NO was associated with the pathogenesis of hearing loss; five papers reported that NO helped prevent and treat the occurrence of hearing loss; and three papers reported that NO was related to NO and hearing loss, but the relationship was not yet clear or related to the progression or treatment of hearing loss (Figure 3).

### 3.1. Studies Investigating the Role of Increased NO in the Pathogenesis of Hearing Loss

NO functions as a double-edged sword. At low concentrations, it plays a role in regulating vascular tone and neurotransmission. However, when produced in excess, NO shifts from a beneficial neuromodulator to a neurotoxic agent. Excessive NO production may result from nNOS activation caused by continuous stimulation of excitatory amino acid receptors—particularly those that mediate glutamate toxicity—or as a result of iNOS induction by stimuli such as endotoxins or cytokines. High concentrations of NO are causally associated with cochlear dysfunction, and numerous studies have reported that excessive NO exacerbates hearing loss.

#### 3.1.1. NO and NO Donor/NO-Producing Compounds

##### S-Nitroso-N-Acetylpenicillamine

NO is a free radical known to play an important role in the development of mesenchymal exudate, but its impact on cochlear function and NO levels in the inner lymph is not clear. A study investigated the effects of nitric oxide (NO) on cochlear function and inner ear fluid concentration, focusing on its potential role in otitis media [13]. In this study, the NO donor compound, S-nitroso-N-acetylpenicillamine (SNAP), was applied to the round window membrane (RWM) of 18 adult chinchillas, and the auditory brain stem response (ABR) threshold was measured before and hourly for 8 h after SNAP application. Two hours after SNAP application, samples of the inner lymph were collected and analyzed for total nitrate and nitrite—end products of NO metabolism. Compared with controls, animals in the experimental group showed significant increases in ABR threshold and nitrate/nitrite in the inner lymph after 5 h. These results suggest that NO in the middle ear can pass through the RWM into the inner ear, contributing to sensorineural hearing loss (SNHL).

##### SNAP-1/3-Morpholinosydnonimine

In a separate study examining direct effects of NO on the morphology of outer hair cells (OHCs) in the cochlea [14], OHCs were collected from the cochlea of chinchillas (*n* = 58) and exposed to the NO-releasing compounds, S-nitroso-N-acetyl-L-penicillamine (1–1.5 mg/mL; Experimental Group 1) or 3-morpholinosydnonimine (SIN-1; 1–1.5 mg/mL; Experimental Group 2). Animals in both experimental groups exhibited ballooning and significant shortening of OHCs compared with the control group (*p* < 0.01). These findings indicate that NO exposure induces irreversible morphological damage in isolated cochlear OHCs, suggesting the possible involvement of NO in the development of SNHL associated with chronic otitis.

##### Acoustic Trauma or Noise

In a study conducted to investigate the role of endogenous NO in temporary threshold shifts (TTS) caused by acoustic trauma [15], healthy, adult, male, Hartley albino guinea pigs (*n* = 34) were exposed to broadband white noise at 105 ± 2 dB sound pressure level (SPL) for 10 min to induce a TTS. Animals were then divided into six groups (N-1 to N-6) based on survival time (0, 1, 2, 3, 7, 28 days), and their auditory brainstem response (ABR) thresholds were measured. After noise exposure, there was an immediate shift in ABR threshold (mean change, 16.2 dB) that was significantly correlated with NO concentration (*p* < 0.001). Compared with the unexposed control condition, the NO concentration nearly tripled immediately after noise exposure. ABR thresholds returned to baseline by the second day post-exposure, whereas NO levels remained elevated (~2-fold). By day 7 after exposure, the NO concentration was not different from that of unexposed control animals. These findings suggest that endogenous NO is produced in response to noise-induced TTS, and that its concentration is closely associated with hearing loss.

Another study explored the potential of photobiomodulation (PBM) therapy to mitigate NIHL and its association with NO signaling [16]. In these experiments, male Sprague-Dawley rats (*n* = 69) were exposed to 1-octave-band noise centered at 4 kHz at 121 dB for 5 h. Following exposure, an 808 nm diode laser beam was applied to the right ear of rats in the PBM treatment group for 30 min daily for 5 days. ABR measurements revealed significantly faster recovery of auditory function in the PBM-treated group on days 4, 7, and 14 after noise exposure compared with the untreated group. Immunofluorescence analyses showed reduced expression of iNOS and cleaved caspase-3 in OHCs of the PBM group compared with the non-treated group, suggesting reduced oxidative stress and apoptosis. In contrast, immunofluorescence and Western blot analyses revealed increased activation of NF-κB, a key upstream regulator of iNOS, in cochlear tissues of the PBM group compared with naive and non-treatment groups (*p* < 0.01). These data suggest that PBM may protect against NIHL by modulating NF-κB activity and reducing iNOS-mediated oxidative stress and caspase-3-dependent apoptosis.

##### Gentamicin

Gentamicin, an important treatment option for Meniere’s disease, has been reported to cause cochlear destruction accompanied by an increase in NO and free oxygen in various animal models, even with interval dosing. In a study that investigated the role of NO in gentamicin-induced hearing loss [17], guinea pigs (*n* = 24) received a single intratympanic injection of gentamicin (10 mg/kg body weight), and ABR and NO production were measured over time. NO production increased after treatment, and ABR thresholds rose by 7.0 dB ± 6.7 SD on day 1, 21.3 dB ± 11.3 SD on day 2, and 32.7 dB ± 14.9 SD on day 7. Levels of NO_2_, a stable oxidative product of NO, also increased along the outer wall. These findings suggest a correlation between NO production and hearing threshold elevation, supporting the hypothesis that increased NO contributes to gentamicin-induced cochlear toxicity and hearing impairment.

#### 3.1.2. NO Inhibitors

##### Platelet-Activating Factor and L-NAME

Platelet-activating factor (PAF) in middle ear exudates causes cochlear hair cell damage, resulting in hearing loss. In a study exploring the role of NO in PAF-induced cochlear damage [18], the therapeutic potential of the NOS inhibitor, NG-nitro-L-arginine methylester (L-NAME), which is known to aid in the treatment of sensory nerve dysfunction caused by chronic middle ear inflammation, was evaluated in fatty-eye and healthy guinea pigs. Experimental animals were divided into four groups: PBS (untreated control), PAF, PAF antagonist (WEB 2170), and L-NAME. Application of PAF directly to the RWM produced dose-dependent increases in ABR threshold, raising ABR after 3 h by 6.5, 19, and 23 dB at 10, 20, and 40 μg PAF, respectively. Compared with untreated controls, animals in the PAF group showed a significant increase in ABR thresholds and clear evidence of cochlear hair cell damage. Immunohistochemical analyses showed strong iNOS expression in the cochlea in the PAF group, and weaker expression in the PBS, WEB 2170, and L-NAME groups. These results suggest that PAF-induced hearing loss resulting from cochlear hair cell damage may be mediated by NO production via iNOS, and suggest that PAF antagonists and NOS inhibitors like L-NAME could have therapeutic value in preventing SNHL associated with chronic otitis media.

##### L-NAME

The NOS inhibitor, L-NAME, is known to suppress NO production and may help prevent damage resulting from acoustic trauma. In a study investigating the protective effects of L-NAME against noise-induced cochlear damage [19], 70 male rats (300–250 g) were divided into four groups: two experimental groups, which received L-NAME 2 days prior (Group I) or immediately before (Group III) noise exposure (octave-band noise at 115 dB SPL for 5 h), and two control groups, a sham-treated group with no noise exposure or L-NAME treatment (Group II) and a noise-exposed group without L-NAME (Group IV). ABR threshold shifts were significantly larger in Group III than in Groups I and IV, and NO levels were also elevated in Group III compared to Group IV. However, cochlear damage caused by noise exposure was significantly reduced in animals treated with L-NAME compared with untreated noise-exposed controls. These findings suggest that inhibition of NOS through L-NAME administration can mitigate NIHL and may offer a therapeutic approach for protecting sensory function in the cochlea.

##### Steroids

Otitis media with effusion (OME) is a common pediatric disease characterized by fluid accumulation in the middle ear that can lead to SNHL if it becomes chronic. NO, a free radical that is known to regulate cell proliferation, cell death, and angiogenesis, is an inflammatory mediator of OME that previous studies have shown can induce SNHL through OHC cytotoxicity. The possibility of using glucocorticoids in the treatment of OME was investigated in a study that tested whether dexamethasone, fluticasone propionate (FP), or rimexolone was able to protect against SNHL in chinchillas (*n* = 53) by reducing NO concentration in the middle ear exudate [20]. All three glucocorticoids significantly reduced NO concentration in the middle ear when administered at 1%; however, only FP produced a significant effect at 0.1%, although there was a trend toward a reduction with the other two agents. These findings suggest that glucocorticoid therapy may lower NO concentration in middle ear exudates and offer protection against NO-induced SNHL.

##### Ascorbic Acid

NIHL has been linked to nitrosative stress and cell destruction resulting from increased NO production in the inner ear. In animal models, ascorbic acid supplementation has been shown to mitigate the severity of hearing loss, suggesting the value of antioxidant supplementation in treating hearing loss. In a study investigating the ability of ascorbic acid to reduce NO levels in inner ear tissues [21], researchers measured local NO production in the organ of Corti and outer wall of the cochlea in male guinea pigs (*n* = 54) 6 h after noise exposure. Animals were provided low-dose (25 mg/d per kg body weight) or high-dose (525 mg/d per kg body weight) dietary ascorbic acid for 7 days prior to exposure to noise at a 90 dB SPL for 1 h. After noise exposure, NO production was significantly reduced in the outer wall and slightly decreased in the organ of Corti in guinea pigs receiving high-dose ascorbic acid treatment. These findings suggest that oral supplementation with ascorbic acid, a natural radical scavenger, reduces NO production in the inner ear in a noisy environment.

##### Taurine

Building on evidence that oxidative and nitrosative stress contribute to drug-induced ototoxicity, one study investigated the potential protective role of taurine against aminoglycoside-induced ototoxicity and the involvement of iNOS in this process [22]. In these experiments, guinea pigs (*n* = 40) were given a single intravenous (i.v.) dose of gentamicin (100 mg/kg), followed by furosemide (90 mg/kg, i.v.). Within 3 days, animals developed severe hearing loss in association with an increase in iNOS expression in the cochlea. Pre-treatment with taurine, which, like aminoguanidine, is a selective iNOS inhibitor, decreased iNOS expression and prevented hearing impairment. These results suggest that taurine exerts a protective effect against acute gentamicin/furosemide ototoxicity through down-regulation of iNOS expression in the cochlea.

##### Astragaloside IV

Astragaloside IV, a major active component of Astragalus membranaceus, is widely used in the treatment of diseases in China owing to its antioxidant properties. In a study designed to test whether Astragaloside IV can reduce acute cochlear injury [23], researchers investigated the effects of orally administered Astragaloside IV on impulse noise-induced damage in guinea pigs (*n* = 36). Astragaloside IV significantly reduced ABR defects, hair cell damage, iNOS expression, and reactive nitrogen species (RNS) formation. These results suggest that the beneficial effects of Astragaloside IV on impulse noise-induced healing loss may be attributable to its ability to suppress iNOS and prevent the formation of RNS.

#### 3.1.3. eNOS Polymorphisms

SSNHL, defined as a hearing loss of 30 dB or more at three or more consecutive frequencies, is a multifactorial condition with an unclear etiology and is, therefore, considered idiopathic. In a study performed by our group involving 77 SSNHL patients and 100 controls [24], we investigated the association between the eNOS protein polymorphism, Glu298Asp, and the VDR gene FokI polymorphism and SSNHL in an Iranian population. We found a significant association between the genotype frequency of the eNOS Glu298Asp polymorphism and SSNHL, with the variant occurring more frequently in patients than in healthy controls (*p* = 0.01). The TT genotype was also the most common genotype in the patient group, where it was significantly increased compared with the control group (TT vs. GT + G, OR = 3.5; 95% CI = 1.18–11.79). In contrast, an analysis of the frequency of the VDR gene FokI polymorphism showed no significant association with SSNHL. These findings demonstrate a significant association between the eNOS Glu298Asp polymorphism and SSNHL and suggest that the TT genotype might be a risk factor for SSNHL.

NOS3 gene variants are associated with an increased risk of sudden SSNHL, suggesting that impaired nitric oxide production may contribute to hearing loss by reducing blood flow to the ear. Specifically, the NOS3 (rs1799983) polymorphism is significantly associated with an increased risk of SSNHL, with an odds ratio (OR) of 2.108 after adjusting for age and sex. Similarly, the Cav1 (rs3840634) polymorphism shows a significant association with an increased risk of Ménière’s disease, with an OR of 1.849 after adjusting for age and sex [25]. These significant associations between specific polymorphisms—NOS3 for SSNHL and Cav1 for Ménière’s disease—and the risk of developing these conditions suggest a potential role for genetic factors in their pathogenesis. These results indicate that these genetic markers may be crucial for understanding the etiology and developing potential treatment strategies for SSNHL and Ménière’s disease (Table 1).

### 3.2. Studies Suggesting That Increased NO Prevents or Mitigates Hearing Loss 

#### 3.2.1. L-Arginine and NO Production

NO plays a crucial role in regulating various physiological processes and shows potential protective effects against hearing loss. L-arginine, a physiological precursor of NO, has been shown to significantly reduce reactive oxygen species (ROS) accumulation in outer hair cells (OHCs) and attenuate noise-induced hearing loss (NIHL) in CBA/J mice. This protective effect is attributed to NO’s ability to enhance S-nitrosylation of pyruvate kinase M2 (PKM2), redirecting glucose metabolism towards the pentose phosphate pathway and increasing the production of reducing agents like NADPH and GSH, which prevent oxidative damage [26].

#### 3.2.2. eNOS and Intrinsic Protection

eNOS is implicated in noise-induced NO production in the cochlea. A study on guinea pigs demonstrated a rapid increase in eNOS immunoreactivity following noise exposure, suggesting an intrinsic protective mechanism in cochlear structures. This rapid upregulation indicates a previously unrecognized role of eNOS in protecting against ototoxic damage [27].

#### 3.2.3. nNOS Deficiency and Hearing Impairment

Mutations in the NOS1 gene, associated with nNOS, have been linked to hearing and other sensory deficits. Studies in Nos1-deficient mice reveal hearing impairments, suggesting that nNOS plays a critical role in auditory function and that its deficiency contributes to hearing defects [28].

#### 3.2.4. NO in Tinnitus and Auditory Modulation

Existing studies have suggested that NO levels in the central cochlear nucleus (VCN) increase in the setting of noise-induced tinnitus, potentially increasing central gain to compensate for reduced input from the cochlea. In a study designed to investigate this mechanism [29], 26 guinea pigs were exposed to noise; of these, 12 were confirmed to exhibit evidence of tinnitus. These results suggest that NO contributes to increasing the gain on stimulus-induced neuronal activity in tinnitus, although other factors are likely responsible for the increase in spontaneous activity. Furthermore, these animal studies underscore the dual role of NO in both central and peripheral auditory pathways, highlighting its potential contribution to modifying neuronal gain and mitochondrial function in noise-induced hearing conditions like tinnitus [30]. This interaction between NO and mitochondrial dysfunction presents a potential target for therapeutic intervention aimed at mitigating the auditory damage and aberrant neural activity associated with tinnitus.

#### 3.2.5. iNOS and Age-Related Cochlear Protection

The role of iNOS in preventing age-related cochlear degeneration was demonstrated in studies with Nos2-knockout mice, which exhibited early-onset hearing impairment. Upregulation of iNOS appears to protect against cochlear regression, highlighting its importance in maintaining auditory health with aging [31] (Table 2).

### 3.3. Studies Suggesting That Increased NO Is Associated with the Development of Hearing Loss, but with Uncertain or Inconclusive Evidence

Despite evidence showing that NO and hearing loss are related, it is not yet clear whether the relationship is positive or negative. Three studies in particular highlight the need for more research on the subject.

#### 3.3.1. NOS Isoforms and Cholesteatoma

**Figure 4:**
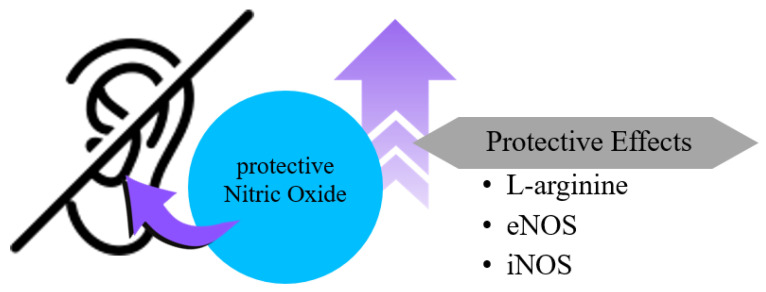
The mechanisms by which increased NO prevents hearing loss involve substances such as L-arginine, eNOS, and iNOS, which contribute to the reduction or prevention of hearing loss by increasing NO levels.

Cholesteatoma is a middle ear disease characterized by inflammatory bone resorption, which primarily results in conductive hearing loss and vestibular dysfunction. Conductive hearing loss occurs because the cholesteatoma disrupts the normal transmission of sound through the middle ear due to damage or erosion of the ossicles, the tiny bones responsible for sound conduction. This bone resorption is thought to be caused by aberrant activation of osteoclasts. A study examining the temporal expression of NOS isoforms in a murine model of cholesteatoma (*n* = 18 mice), produced by keratin implantation in the middle ear, showed expression of all three NOS isoforms [32]. NOS1 and NOS3 expression was low to very low during the study period and was unchanged by keratin implantation. However, NOS2 (iNOS) was significantly upregulated following keratin implantation. Interferon γ (IFNγ) alone promoted nitrite production in vitro, whereas interleukin (IL)-1β and tumor necrosis factor alpha (TNFα) did not. Combined treatment with IL-1β, TNFα, and IFNγ synergistically increased nitrite production, an effect that was blocked by aminoguanidine (AG), a selective NOS2 inhibitor. Despite this, in vivo AG treatment did not significantly alter osteoclast activity in the cholesteatoma-induced bone-resorption model. These results indicate that, although only NOS2 among the three NOS isoforms is significantly upregulated in response to keratin, its selective inhibition does not affect osteoclast activity, suggesting that osteoclast-dependent inflammatory bone resorption in cholesteatoma is mediated by NOS-independent pathways (Figure 4).

#### 3.3.2. NO Donors and Inhibitors

The effects of NO donors (SIN-1 and SNOG) and the NOS inhibitor L-NAME on central cochlear nucleus activity were examined in guinea pigs. The NO donors caused variable changes in neuronal firing rates, while L-NAME consistently increased driven firing rates in 18% of recorded units without affecting spontaneous rates. These findings imply that abnormal NO levels may modulate neural gain, but the study did not reach definitive conclusions about NO’s role in hearing loss [33].

#### 3.3.3. NO-GC Knockout Models

NO activates soluble guanylate cyclase (NO-GC), which is involved in cGMP-mediated signaling pathways. In NO-GC1-KO and NO-GC2-KO mice, lacking key NO-sensitizing GC isoforms, there were no effects on outer hair cell function or auditory thresholds before or after noise exposure. Furthermore, long-term pharmacological stimulation of NO-GC had no impact on hearing, suggesting that NO signaling through these pathways does not contribute to hearing loss development [34].

#### 3.3.4. NOS Inhibitor

Inhibitors of nitric oxide synthase (NOS) such as N-nitro-l-arginine (NNLA), 7-nitroindazole (7NI), and aminoguanidine were evaluated for their potential protective effects against acoustic injury. However, these NOS inhibitors did not produce any significant changes in the auditory brainstem response (ABR) threshold shifts, nor did they prevent the loss of outer hair cells [35]. This lack of effect suggests that NOS inhibitors do not confer protective benefits against cochlear damage induced by high sound levels. These findings suggest that the data imply that nitric oxide pathways may not play a significant role in mediating acoustic injury, in the context of NOS inhibition, highlighting the need to explore alternative molecular targets for effective intervention (Table 3).

## 4. Limitations

This study has several limitations that should be considered when interpreting the findings: One limitation is the diversity of subjects included in the study. Our review encompasses both animal and human studies, introducing variability. Different animal species, such as mice, rats, chinchillas, and guinea pigs, are involved, and each may respond differently to nitric oxide (NO) and its effects on hearing. These differences can lead to conflicting results across studies. Another limitation is the variation in research methods used. Some studies examine the effects of acute exposure to noise or substances, while others investigate chronic exposure. These differences in experimental design can affect outcomes and interpretations of NO’s role in hearing, making direct comparisons challenging. The studies also vary in terms of the specific genes they target. Each study focuses on different genes related to NO production, which can lead to varying interpretations and influence the consistency of results. Additionally, the effects of different nitric oxide synthase (NOS) isoforms, such as nNOS, eNOS, and iNOS, add complexity, as each isoform may play a unique role in auditory processes. The variety of intervention substances used is another factor contributing to inconsistent findings. Different substances can affect the degree and amount of NO or NOS expression, making it important to consider these interventions when comparing results. A significant challenge is the ambiguity in determining causality between NO/NOS expression and hearing loss. It is unclear whether changes in NO/NOS levels lead to hearing loss or are a result of it, complicating the interpretation of results. Lastly, the exact amount of NO and its impact on hearing were not consistently quantified across studies. Depending on its concentration, NO may have protective or detrimental effects on hearing. This underscores the importance of precise quantification in future research to better understand NO’s role. By addressing these limitations, we aim to provide context for the variability observed in study outcomes and underscore the importance of further research to clarify NO’s role in hearing loss.

## 5. Conclusions

Our review of 23 studies reveals the complex and dual role of NO in auditory physiology and pathology. NO is an essential signaling molecule that plays a critical role in maintaining normal auditory functions. It regulates blood flow, neurotransmission, and ion channel activity within the cochlea, ensuring an adequate supply of oxygen and nutrients through vasodilation, which is vital for preserving auditory health. However, the review also highlights that excessive or dysregulated NO production can lead to ototoxicity and hearing loss. When NO levels become elevated, particularly through iNOS, it acts as a potent oxidant. This overproduction can cause DNA damage, protein denaturation, and apoptosis of OHCs and neurons, contributing to hearing impairment. Interestingly, the reviewed studies also show that NO can have protective effects in certain contexts. For example, NO has been reported to mitigate noise-induced hearing loss by acting as an antioxidant, reducing reactive oxygen species, and enhancing cochlear resilience. This protective role underscores NO’s potential as a therapeutic target, where modulating its levels could prevent or alleviate hearing loss. The findings suggest that the effects of NO on hearing depend significantly on its concentration and the specific auditory context. While most studies indicate that excessive NO exacerbates hearing loss, some highlight its protective benefits, emphasizing the importance of balanced NO production. Looking forward, targeting NO pathways offers promising therapeutic strategies for managing hearing loss. However, further research is needed to standardize experimental approaches, clarify the conditions under which NO is beneficial or harmful, and develop precise interventions. By understanding and leveraging NO’s dualistic nature, we can advance towards effective treatments for hearing loss. This nuanced perspective on NO’s role in auditory health and pathology offers valuable insights into potential therapeutic approaches and highlights the need for continued research in this area.

## Figures and Tables

**Table 1:** Negative relationship between NO and hearing loss.

Author [Reference]	Study Design	Species and/or Sample	Detection Method	Causative Agent	Target Gene(s)	Results/Conclusion
Jung T.T. et al., 2003 [13]	Animal study	58 chinchillas	A NO derivative was used to generate NO externally, and the corresponding change in the form of OHC was detected using the IMAGE Pro-Plus program.	SNAP	iNOS	OHCs exposed to either a standard bathing solution or sodium nitrite (Control Groups 1 and 2) did not exhibit any significant changes in cell shape or length. However, cells exposed to NO donors, such as SNAP or SIN-1, displayed ballooning and significant shortening in mean cell length (*p* < 0.01). /Exposure to NO induces irreversible morphological changes in isolated OHCs, suggesting that NO radicals may play a role in the development of SNHL as a consequence of chronic otitis media.
Heinrich U.R. et al., 2008 [14]	Animal study	24 guinea pigs	Chemiluminescence	SNAP	eNOS	One day after injection, gentamicin caused a 1.34-fold increase in the production of NO_2_ (a stable oxidative product of NO) in the organ of Corti compared with control conditions (physiological saline). NO_2_ concentrations in the organ of Corti remained similar 2 and 7 days after gentamicin application. NO_2_ levels also significantly increased by a factor of 3.24 in the lateral wall 2 days post-injection, reaching a mean concentration of 874 nM. Gentamicin application led to a shift in the hearing threshold beginning on the second day after application. /Hearing impairment was correlated with an increase in NO_2_ content in the lateral wall. NO_2_ production increased slightly in the organ of Corti as early as day 1 after gentamicin injection.
Chen Y.S. et al., 2005 [15]	Animal study	34 guinea pigs	NO Analyzer 280A	Acoustic trauma or noise	iNOS	An average 16.2 dB threshold shift was found immediately after noise exposure. The threshold returned to the pre-noise-exposed level on the second day post-exposure. Compared to unexposed control animals, the NO concentration increased nearly threefold immediately following noise exposure and decreased to twofold when the hearing threshold returned to the pre-noise-exposed level. /The increase in NO concentration after acoustic trauma is associated with hearing loss, indicating that a high concentration of NO is neurotoxic for cochlear function.
Atsushi Tamura et al., 2016 [16]	Animal study	69 rats	Immunofluorescence	Acoustic trauma or noise	iNOS	Immunofluorescence image analysis of NF-κB, an upstream regulator of iNOS, revealed greater activation in the PBM group compared with naïve and non-treatment groups. Western blot analysis of NF-κB also showed stronger activation in cochlear tissues in the PBM group compared with naïve and non-treatment groups (*p* < 0.01 each). /PBM activates NF-κB, providing protection against iNOS-triggered oxidative stress and caspase-3-mediated apoptosis that accompanies NIHL.
Jonathan B. Hanson et al., 2003 [17]	Animal study	18 chinchillas	Colorimetric assay	Gentamicin	iNOS	Comparing the mean ABR threshold measurements in the SNAP-operated (SNAP-OP) group with the SNAP non-operated (SNAP Non-OP) control group (contralateral) from 1 to 8 h following the application of a fresh SNAP solution on the round window membrane, there was a significant increase (*p* < 0.05) in the ABR threshold after 3 h in the experimental group compared to the control group. In the operated ears, levels of NO metabolites (NO_2^−^_ + NO_3^−^_) in the perilymph were approximately three times higher (*p* = 0.02) than those in the non-operated ears. /While NO seems to play a crucial role in the development of middle ear effusions and can lead to cochlear damage, its impact on the inner ear as it moves from the middle ear space remains undocumented.
Chung-Ku Rhee et al., 2003 [18]	Animal study	70 guinea pigs	Immunohistochemistry	PAF and NAME	iNOS	The ABR threshold and cochlear hair cell damage were notably higher in the PAF treatment group compared to the PBS control group. In the group treated with a PAF-antagonist, the threshold increased to 7 dB SPL at 3 h, fluctuating between 7 and 11 dB SPL throughout the 24 h period. /Application of PAF to the RWM resulted in hearing loss and damage to cochlear hair cells. However, the use of PAF-antagonists and L-NAME effectively prevented PAF-induced hearing loss and suppressed iNOS expression in the cochlea.
Charles Pudrith et al., 2010 [19]	Animal study	53 chinchillas	Griess Reagent Assay	L-NAME	iNOS	At a 0.1% concentration, the three glucocorticoids—dexamethasone, fluticasone propionate, and rimexolone—demonstrated a numerical decrease in NO levels within middle ear effusions, with only fluticasone propionate achieving a statistically significant reduction. At a higher concentration of 1.0%, all three glucocorticoids markedly lowered NO concentrations, with an average reduction of 55.3%. /Glucocorticoid therapy effectively lowers NO levels in middle ear effusions within a lipopolysaccharide-induced otitis media model, implying that glucocorticoids might offer protection against SNHL by curbing NO-mediated ototoxicity.
Heinrich U.R. et al., 2008 [20]	Animal study	54 guinea pigs	The amount of NO produced was indirectly evaluated by detecting nitrite, a stable oxidative metabolite of NO, by chemiluminescence.	Steroids	iNOS	Treatment with ascorbic acid led to a dose-dependent decrease in hearing thresholds following noise exposure. When administered in high doses, ascorbic acid notably reduced NO production in the lateral wall post-noise exposure and showed a tendency to lower NO production in the organ of Corti. /Oral supplementation with the natural radical scavenger ascorbic acid diminishes the rate of NO production in the inner ear under noisy conditions, supporting its protective role against inner ear damage.
Diao M. et al., 2007 [21]	Animal study	70 guinea pigs	NO assay kit	Ascorbic acid	iNOS	Immediate pre-treatment with L-NAME (Group III) led to significantly greater outer hair cell (OHC) loss, threshold shifts, and NO levels when compared with 2-day pre-treatment (Group I) and noise-exposed animals without L-NAME (Group IV). The increase in NO levels in the cochlea due to noise was significantly reduced by L-NAME compared to Group III (*p* < 0.001). /L-NAME provides protection against cochlear damage from acoustic trauma by decreasing NO production.
Liu H.Y. et al., 2008 [22]	Animal study	40 guinea pigs	Western blot, immunofluorescence	Taurine	iNOS	In an in vivo model of gentamycin/furosemide-induced ototoxicity, iNOS expression in the cochlea was significantly increased three days following treatment, correlating with severe hearing loss. Pretreatment with taurine or aminoguanidine, both of which are selective iNOS inhibitors, resulted in the downregulation of iNOS and prevented hearing damage. /Taurine offers protection against acute ototoxicity from gentamycin/furosemide, potentially by downregulating iNOS expression in the cochlea.
Min Xiong et al., 2011 [23]	Animal study	36 guinea pigs	Immunohistochemistry	Astragaloside Ⅳ	iNOS	Astragaloside IV significantly mitigated ABR deficits, hair cell damage, iNOS expression, and RNS formation in a model of impulse noise-induced hearing loss. /The protective effect of Astragaloside IV against impulse NIHL may be due to its capacity to inhibit iNOS and prevent RNS formation.
Nasrin Yazdani et al., 2018 [24]	Case study	77 patients	eNOS genetic analysis	NO polymorphisms	eNOS	A statistically significant correlation was identified between the genotype frequencies of the eNOS protein polymorphism, Glu298Asp, and the presence of SSNHL in the patient group compared to healthy controls. The TT genotype was notably more prevalent in the patient group than in the control group. /The eNOS protein polymorphism, Glu298Asp, shows a significant association with SSNHL in an Iranian population, suggesting that the TT genotype could be considered a risk factor for SSNHL.
Teranishi et a., 2013 [25]	Clinical study	4160 participants	Polymerase Chain Reaction (PCR), Genotyping	NO polymorphisms	eNOS	Certain genetic variations, particularly in genes related to the body’s free-radical processes, are significantly associated with an increased risk of idiopathic sudden SSNHL and Ménière’s disease. Specifically, a variant in the NOS3 gene, which affects nitric oxide production, was notably linked to a higher risk of SSNHL. This was determined by comparing patients with these conditions to large control groups, highlighting the influence of these genetic factors. /Genetic differences influencing the free-radical process may play a role in the development of SSNHL and Ménière’s disease. These insights could pave the way for more personalized treatment approaches and help identify individuals at greater genetic risk for these conditions. The study also underscores the need for additional research to further explore these genetic influences and confirm the results in larger populations.

Abbreviations: NO, nitric oxide; iNOS, inducible nitric oxide synthase; OHC, outer hair cell; PAF, platelet-activating factor; SPL, sound pressure level; NOS, nitric oxide synthase; RWM, round window membrane; eNOS, endothelial nitric oxide synthase; NF-κB, nuclear factor kappa-light-chain-enhancer of activated B cells; L-NAME, N-nitro-L-arginine methyl ester; RNS, reactive nitrogen species; SSNHL, sudden sensorineural hearing loss. Note: The “/” denotes a separator between sections (Results/Conclusion).

**Table 2:** Positive relationship between NO and hearing loss.

Author [Reference]	Study Design	Species and/or Sample	Detection Method	Causative Agent	Target Gene(s)	Results/Conclusion
Haidi Yang et al., 2021 [26]	Animal study	30~40 mice	Colorimetric NO assay	L-argininge	eNOS	The cytosol of OHCs, IHCs, spiral ganglion cells, and stria vascularis cells exhibited strong immunostaining for eNOS. In response to noise exposure, NO levels in the cochlea increased as a compensatory mechanism. Supplementing with L-arginine further elevated NO levels and protected against NIHL in mice. Noise exposure led to a significant reduction in wave amplitude (from 60 to 80 dB SPL) compared to controls, a decrease that was mitigated by L-arginine supplementation. /NO helps to minimize noise-induced ROS buildup in the cochlea and reduces NIHL in mice.
Heinrich U.R. et al., 2019 [27]	Animal study	24 guinea pigs	Immunohistochemistry, immunoelectron microscopy	eNOS	eNOS	Exposure to 90 dB for 1 or 2 h caused a rapid increase in eNOS immunostaining intensity in almost all cochlear regions compared to controls, aligning with previous findings that eNOS regulation occurs at transcriptional, translational, and post-translational levels. /Similarly to iNOS, eNOS can be upregulated, with its cochlear expression increasing in response to noise exposure. Additionally, the reticular lamina, which forms the endolymph-perilymph barrier at the apical side of the organ of Corti, participates in a swift, intrinsic cochlear otoprotective mechanism.
Konstantina Chachlaki et al., 2022 [28]	Animal study	Human and mice	Live-cell imaging using NO sensor (FlincG3), fluorometric nitrate assay	nNOS	iNOS	NOS1 was transiently expressed by GnRH neurons in the nasal region of both humans and mice. In mice, a deficiency in Nos1 led to abnormalities in sexual maturation, as well as impairments in olfaction, hearing, and cognitive functions. /The absence of timely NOS1 activity results in a GnRH deficiency, contributing to lifelong sensory and intellectual comorbidities in humans and mice. By addressing deficits in sexual maturation, olfaction, and cognition in Nos1-deficient mice within a critical period, NO treatment shows therapeutic promise for humans.
Adam Hockley et al., 2020 [29]	Animal study	26 guinea pigs	Iontophoresis	NO	nNOS	In the context of noise-induced tinnitus, a higher percentage of neural units responded to externally applied NO in both tinnitus (56%) and non-tinnitus groups (71%) compared to the control group (24%). Within the tinnitus group, endogenous NO increased the driven firing rate in 37% (7 out of 19) of neurons, seemingly restoring the mean driven firing rate to control levels via a mechanism involving NMDA receptors. Conversely, in the non-tinnitus group, endogenous NO enhanced the driven firing rate in only 5% (1 out of 22) of neurons and did not impact the driven firing rate in the control group. /NO may play a role in augmenting the gain on neurally driven activity, although other factors also contribute to the rise in spontaneous activity.
Shi et al., 2007 [30]	Animal study	80 albino guinea pigs	Fluorescent dyes, Immunocytochemistry	NO		NO was predominantly localized in the mitochondria of OHCs in the cochlea. Exposure to loud noise and treatment with an NO donor both resulted in reduced mitochondrial membrane potential and increased NO levels. Additionally, there was an increase in nitrotyrosine, indicating the formation of peroxynitrite, a reactive nitrogen species, which suggests oxidative stress within the mitochondria. /NO plays a crucial role in regulating the energy status of OHCs and contributes to cellular damage under stress conditions like loud noise exposure. This interaction between NO and mitochondria could be a key factor in the pathology of NIHL, highlighting potential therapeutic targets to mitigate hearing damage by focusing on mitochondrial health and NO pathways.
Daniel Labbé et al., 2016 [31]	Animal study	30~50 mice	Immunohistochemistry	iNOS	iNOS	A study examining the role of iNOS (NOS2) in age-related cochlear regression revealed that NOS2 is upregulated in wild-type (WT) mice after 6 months of age. Nos2 knockout (NOS2-KO) mice displayed slightly impaired hearing in adulthood, with an accelerated decline in hearing as they aged, accompanied by increased nitrotyrosine formation and loss of OHCs. /The induction of NOS2 serves as a protective mechanism against age-related cochlear degeneration.

Abbreviations: eNOS, endothelial nitric oxide synthase; NO, nitric oxide; NIHL, noise-induced hearing loss; SPL, sound pressure level; ROS, reactive oxygen species; iNOS, inducible nitric oxide synthase; NOS, nitric oxide synthase; GnRH, gonadotropin-releasing hormone; KO, knockout; WT, wildtype; NMDA, N-methyl-D-aspartic acid; OHC, outer hair cell; IHC, inner hair cell. Note: The “/” denotes a separator between sections (Results/Conclusion).

**Table 3:** NO and hearing loss have an uncertain or no connection.

Author [Reference]	Study Design	Species and/or Sample	Detection Method	Causative Agent	Target Gene(s)	Results/Conclusion
Jung, Jae Y. et al., 2004 [32]	Animal study	18 mouse model	Colorimetric assay, in vitro osteoclast culture method	NOS isoforms	iNOS	In a keratin implant-induced model of cholesteatoma-induced bone resorption, the expression levels of NOS1 and NOS3 remained low or very low throughout the study and did not change in response to the keratin implant. In contrast, NOS2 was upregulated due to the keratin implantation. While AG, a selective NOS2 inhibitor, reduced nitrite production in vitro, it did not affect the osteoclast response in vivo. /Although NOS2 was upregulated in vivo in this model, and AG suppressed nitrite production in vitro, the lack of effect of AG on the osteoclast response in vivo suggests that bone loss in this model is driven by a NOS-independent mechanism.
Adam Hockley et al., 1997 [33]	Animal study	27 male and female guinea pigs.	Iontophoresis, Immunohistochemistry, sound system response	L-NAME	nNOS	Inhibition of endogenous NO production using L-NAME led to a consistent increase in driven firing rates in 18% of neural units, while having minimal impact on spontaneous rates. This reduction in gain caused by endogenous NO was reflected in the effects of L-NAME on NMDA-evoked excitation, with 30% of units displaying enhanced NMDA-evoked excitation when NO levels were reduced by L-NAME application. Approximately 25% of neurons were nNOS-positive, and the NO they produced can modulate the firing rates of the main principal cells: medium stellates (choppers), large stellates (onset responses), and bushy cells (primary-like responses). /Endogenous NO seems to primarily function in suppressing driven firing rates linked to NMDA channel activity, but it also serves to increase neural gain when there are pathological increases in its production following hearing loss.
Dorit Möhrle et al., 2017 [34]	Animal study	12 KO mice	Fluorescence resonance energy transfer, ABR, and DPOAE, RT-PCR.	NO-GC1, NO-GC2	iNOS	A NO-induced increase in cGMP was observed in real-time within inner hair cells, but not in OHCs. Pharmacological long-term treatment with an NO-GC stimulator modified auditory nerve responses without affecting OHC function or hearing thresholds. Interestingly, NO-GC stimulation worsened the decline of auditory nerve response in older animals but mitigated it in younger animals. /NO-GC2 and, to a lesser extent, NO-GC1 may serve as targets for early pharmacological intervention to prevent auditory fiber loss (synaptopathy). Both isoforms offer selective advantages for hearing function by preserving the functional integrity of auditory nerve fibers during early life rather than in old age.
Murashita et al., 2006 [35]	Animal study	67 female mice	Auditory brainstem response (ABR), cochlear surface preparation	Tempol, 3-aminobenzamide, and nitric oxide synthase inhibitors	NOS isoforms	Tempol, a superoxide anion scavenger, and 3-aminobenzamide (3AB), a PARS inhibitor, both significantly protected the cochlea from acoustic injury by reducing ABR threshold shifts and hair cell loss. The protective effects were dose-dependent. In contrast, NOS inhibitors did not show any protective effects. /The study suggests that ROS and PARS play crucial roles in acoustic injury, as evidenced by the protective effects of tempol and 3AB. The lack of effect from NOS inhibitors implies a lesser role for nitric oxide in such injuries. These findings point to ROS and PARS as potential therapeutic targets, but further research is needed to understand the underlying mechanisms.

Abbreviations: nNOS, neuronal nitric oxide synthase; NO, nitric oxide; iNOS, inducible nitric oxide synthase; NOS, nitric oxide synthase; AG, aminoguanidine; NO-GC, nitric oxide-sensitive guanylate cyclase; PCR, Polymerase Chain Reaction. Note: The “/” denotes a separator between sections (Results/Conclusion).

## Data Availability

Not applicable.
